# Protocol for a prospective observational cohort study of cutaneous leishmaniasis in Ethiopia

**DOI:** 10.3310/nihropenres.13432.1

**Published:** 2023-10-05

**Authors:** Amel Beshir Mohammed, Fewzia Shikur Mohammed, Feleke Tilahun Zewdu, Shimelis Doni Nigusse, Yohannes Hailemichael, Teklu Cherkose, Abebaw Yeshambel Alemu, Eshetu Molla, Kidist Bobosha, Vanessa Yardley, Iris Mosweu, Mirgissa Kaba, Catherine Pitt, Elizabeth Allen, Saba Maria Lambert, Michael Marks, Stephen L. Walker, Endalamaw Gadisa, SHARP collaboration

**Affiliations:** 1 ALERT Hospital, Addis Ababa, Ethiopia; 2 Department of Dermatovenereology, College of Health Sciences, Addis Ababa University, Addis Ababa, Ethiopia; 3 Boru Meda Hospital, Boru Meda, Ethiopia; 4 Department of Epidemiology and Biostatistics, Institute of Public Health, College of Medicine and Health Sciences, University of Gondar, Gondar, Ethiopia; 5 Department of Malaria and Neglected Tropical Disease Research, Armauer Hansen Research Institute, Addis Ababa, Ethiopia; 6 Department of Paediatrics and Neonatal Health Nursing, College of Health Sciences, Debre Tabor University, Debre Tabor, Ethiopia; 7 Department of Infection Biology, Faculty of Infectious and Tropical Diseases, London School of Hygiene & Tropical Medicine, London, UK; 8 Department of Global Health and Development, London School of Hygiene & Tropical Medicine, London, UK; 9 School of Public Health, Addis Ababa University, Addis Ababa, Ethiopia; 10 Department of Medical Statistics, Faculty of Epidemiology and Population Health, London School of Hygiene & Tropical Medicine, London, UK; 11 Department of Clinical Research, Faculty of Infectious and Tropical Diseases, London School of Hygiene & Tropical Medicine, London, UK; 12 Hospital for Tropical Diseases, University College London Hospitals NHS Foundation Trust, London, UK; 13 University College London, London, UK

**Keywords:** Cutaneous leishmaniasis, L. aethiopica, clinical outcomes measure, scar assessment, cost effectiveness, patient reported outcome measures, Ethiopia

## Abstract

**Background:**

Cutaneous leishmaniasis (CL) is a skin neglected tropical disease, with an estimated 40,000 new cases each year in Ethiopia. CL causes ulcers, nodules, and plaques on the skin, and in some instances the destruction of the nasopharyngeal mucosa and cartilage. Some CL lesions may heal spontaneously, whilst other lesions may require therapies which are associated with discomfort, adverse effects, prolonged treatment, and a frequent lack of a complete response. Scarring, a sequela of CL, causes permanent disfigurement and is associated with stigma linked with a reduction in health-related quality of life.

The choice of treatment for CL is based upon factors including the causative species; the number, extent, size, and location of lesions; and the availability of treatments. The development of robust evidence for CL treatment is hindered by a lack of validated and appropriate outcome measures and few data to support hypothesis-generation and trial design. There is a paucity of prospective data with well-defined treatment outcomes for CL caused by *L. aethiopica*.

**Aim:**

The overall aim of this study is to improve the understanding of the health and economic burden of CL.

**Methods:**

We have designed an observational, multi-centre cohort study to examine treatment outcomes for CL in Ethiopia which includes clinical outcomes, laboratory outcomes, patient reported outcome measures, scar assessments and cost effectiveness. We aim to recruit up to 750 participants across two hospital sites. We present here the protocol for this cohort study with a 12-month follow up period for each participant.

**Conclusions:**

These data will inform the design of randomized controlled trials to evaluate new treatment strategies, with appropriate economic evaluations. This will help improve evidence-based guidelines and support evidence-led policy decisions, not only in Ethiopia but also globally.

## Plain English summary

Cutaneous leishmaniasis (CL) is a skin neglected tropical disease, with an estimated 40,000 new cases each year in Ethiopia. CL is transmitted by the bite of a sand fly on exposed areas, often the face, resulting in ulcers, nodules, and plaques on the skin, and in some instances the destruction of the nasopharyngeal mucosa and cartilage. Some CL lesions may heal spontaneously, whilst other lesions may require therapies which are associated with discomfort, adverse effects, prolonged treatment, and a frequent lack of a complete response. Scarring, a sequela of CL, causes permanent disfigurement and is associated with stigma linked with a reduction in health-related quality of life.

The choice of treatment for CL is based upon factors including the causative species; the number, extent, size, and location of lesions; and the availability of treatments. The development of robust evidence for CL treatment is hindered by a lack of validated and appropriate outcome measures and few data to support hypothesis-generation and trial design. There is a paucity of prospective data with well-defined treatment outcomes for CL caused by L. aethiopica. We therefore designed an observational, multi-centre cohort study to examine treatment outcomes for CL in Ethiopia which includes clinical outcomes, laboratory outcomes, patient reported outcome measures, scar assessments and cost effectiveness.

These essential data will inform the design of randomized controlled trials to evaluate new treatment strategies, with appropriate economic evaluations. This will help improve evidence-based guidelines and support evidence-led policy decisions, not only in Ethiopia but also globally.

## Introduction

Up to a million new cases of cutaneous leishmaniasis (CL) occur each year, primarily in the world’s poorest communities ^1^. CL is the most common form of leishmaniasis, which is caused by infection with protozoa of the genus Leishmania, acquired via the bite of a female phlebotomine sandfly. Infection leads to the development of ulcers, nodules, and plaques on the skin. Depending on the infecting *Leishmania* species, spontaneous resolution of infection can take many months or years and medical therapies are associated with discomfort, adverse effects, prolonged treatment, and a frequent lack of complete response ( World Health Organization). Lesions often heal with lifelong scarring or destruction of the nasopharyngeal mucosa causing permanent disfigurement, and associated stigma, and overall psychological morbidity, contributing to a reduction in health-related quality of life (HRQoL) ^2^. These multidimensional morbidities and the often-arduous process of treatment-seeking also impose a substantial economic burden in terms of both costs of care and income loss, which can exacerbate the stress and social impact associated with CL and inhibit access to care ^3– 5^.

The choice of treatment for CL is based upon several factors including the causative species; the number, extent, size and location of lesions; and the availability of treatments. Small lesions not located near a joint or facial structure may be allowed to self-heal or be treated with a topical agent, intra-lesional injection, cryotherapy, or heat therapy. Localised treatments for CL are associated with irritation, pain, discomfort, and in the case of cryotherapy, long-term skin changes ^6^. Large or widespread lesions and those involving the mucosa require systemic treatment ^7, 8^. Despite the large number of cases of CL each year, the evidence for determining the best treatment modality remains limited.

In Ethiopia, CL is a growing public health problem, with an estimated 40,000 new cases each year. Several *Leishmania* species cause CL in Ethiopia; most infections are thought to be due to *L. aethiopica* ^9^, but *L. donovani*, *L. major* and *L. tropica* are also present. *L. aethiopica* is associated with three distinct clinical forms of CL; localised CL (LCL); mucocutaneous leishmaniasis (MCL); and diffuse CL (DCL), which is the most disfiguring and difficult to treat ^9^. Lesions caused by *L. aethiopica* most commonly occur on the face ^10^. The diverse range of clinical phenotypes and presentations makes early diagnosis by community-based health workers difficult. Standard CL treatment in Ethiopia is with intralesional or parenteral sodium stibogluconate (SSG), which may be combined with cryotherapy using liquid nitrogen. There is a particular paucity of prospective data with well-defined treatment outcomes for CL caused by *L. aethiopica*. A systematic review of treatment for CL caused by *L. aethiopica* identified two randomised trials with a total of 26 participants and two prospective, non-randomised studies ^11^. All four studies were assessed to have methodological limitations by the authors. One of the small, randomised trials compared itraconazole with placebo (n=14 participants) ^12^ and the other compared rifampicin, isoniazid and amithiozone with pentamidine (n=12 participants) ^13^. The much larger prospective study reported improvement in various forms of CL: 81% of participants improved with liquid nitrogen cryotherapy and 85% improved with intramuscular SSG (20 mg/kg up to a maximum of 850mg daily) for 30 days ^14^. Another study reported that 21% of participants had “a positive outcome” with intralesional pentavalent antimonial drug meglumine antimoniate (MA) and 47% had “a positive outcome” with systemic MA (20 mg/kg) ^15^.

The development of robust evidence for CL treatment is hindered by a lack of validated and appropriate outcome measures and few data to support hypothesis-generation and trial design. A Cochrane review demonstrated that existing studies used heterogeneous outcome measures which mostly lacked validity, hindering comparison of study results ^16^. A consensus document on “harmonized” clinical research methodologies for LCL (but not MCL or DCL) was published by an international group of experts following meetings organised by WHO and the Drugs for Neglected Diseases initiative ^17^. This publication highlighted the importance of laboratory confirmation with identification of *Leishmania* species in future trials and defined “initial” cure for non-American CL as more than 50% re-epithelialization (if an ulcer) or flattening (if a non-ulcerated lesion) at Day 42; and as 100% re-epithelialization or flattening at Day 90. “Final” cure was defined as 100% re-epithelialization or flattening at Day 180. More recently, open label studies, incorporating these expert-agreed outcomes, were conducted on treatment for CL due to *L. aethiopica*. One study reported physician-determined cure rates of 59.7% for intralesional SSG ^18^ at Day 90, whilst another study reported physician-determined cure rates of 12.5% and 72.7% for miltefosine ^19^ at two study sites at Day 90.

The “harmonized” clinical research methodologies for LCL provided a significant step forward but several gaps remain. No agreed methodologies are available for assessing flattening or re-epithelisation and it remains unclear if outcomes should be measured from the start or end of a course of treatment. The agreed outcome measures are entirely clinical and do not include measures to reflect the outcomes and experiences of affected individuals. The treatment preferences of individuals may be influenced by the frequency of adverse events and the extent to which healing improves their health and psychosocial wellbeing. Appropriate patient reported outcome measures (PROMs) therefore need to be identified and collected alongside clinical measures. Finally, the consensus document recognised that scarring should be assessed as a long-term outcome but did not propose a method for doing so.

Given the scarcity of resources for health, and for the treatment of CL in particular, treatment recommendations should also be informed by an understanding of costs and efficiency. However, cost-effectiveness analyses of alternative treatment strategies for non-American CL have only been conducted in Asia ^20– 23^, none have addressed infection with *L. aethiopica*, and only one has assessed effectiveness using a multidimensional outcome metric ^20^, which is essential for comparing the value of investments across intervention types and diseases. No studies of the economic burden of CL or treatment costs in Africa have been published. 

The Ethiopian Federal Ministry of Health (FMOH) has identified an urgent need for the evaluation of treatments alternatives for all forms of CL. Such evaluation requires better understanding of outcomes, and cost-effectiveness data. We therefore designed an observational, multi-centre cohort study of CL in Ethiopia ^24^.

## Aims & objectives

The overall aim of this study is to improve the understanding of the health, psychosocial and economic burden of CL, and to lay the groundwork for well-designed, randomized controlled trials and economic evaluations of alternative CL treatments in Ethiopia.

### Objectives

1. To assess clinical outcomes in LCL, MCL and DCL in a prospective cohort
2. To assess clinical parameters in LCL, MCL and DCL in a prospective cohort
3. To assess the change in HRQoL associated with CL before and after treatment.
4. To develop a severity scale for CL
5. To understand the lived experiences of individuals with CL attending a referral centre by conducting in-depth interviews
6. To assess household costs of CL, health care provider and societal costs of treating CL
7. To characterise how cell-mediated immune responses associate with treatment responses in CL due to L. aethiopica and Human Leucocyte Antigen (HLA) variation correlate with different clinical phenotypes.

## Methods

### Patient and Public Involvement

Patients with CL were involved in the validation of the translations of PROMs through focus group discussions, cultural adaptation and piloting.

### Study context

The study will enrol individuals receiving treatment for CL at two centres: ALERT Hospital, Addis Ababa and Boru Meda Hospital, South Wollo Zone, Amhara Region. ALERT Hospital serves as both a general hospital for patients in Addis Ababa and a regional and national specialized referral centre for individuals with CL and other skin conditions. The hospital has nine dermatologists and a 20-bed unit for the care of patients with CL and diagnoses approximately 1000 cases of CL managed on an inpatient or outpatient basis each year. Boru Meda serves as the general hospital and specialised referral service for all skin conditions in South Wollo zone. It has two dermatologists, and a 35-bed unit for the management of CL and diagnoses around 700 cases of CL, managed on an inpatient or outpatient basis each year.

### Timelines

Study recruitment began in April 2022 and is expected to continue until the sample size is reached or until the cut-off of September 30th, 2023, whichever occurs first. Follow-up is therefore expected to be completed by September 30th, 2024.

### Study participants and management

Adults and children with CL who are managed as inpatients or outpatients are eligible to participate in the study.

Inclusion Criteria: Participants with laboratory confirmed CL

Exclusion Criteria: Participants who have previously been treated with systemic anti-leishmanial therapy

Participants will be treated using the standard of care for CL at the centre where they are managed and in accordance with the Ethiopian national guideline ^24^ which will be agreed by two clinicians in consultation with the affected individual prior to enrolment. The treatment may be changed if deemed clinically appropriate. In general, if the size of the skin lesion is 4cm in diameter of smaller and not near a mucosal site the first line treatment is most frequently local therapy (cryotherapy and/or intralesional SSG). In general, if the diameter of the skin lesion is greater than 4 cm and/or near or involving a mucosal surface the initial therapy is usually intramuscular SSG for 28 days with or without adjunctive cryotherapy. Participants will be assessed on day 42 and further local or systemic therapy may be offered depending on the degree of clinical response.

We will classify CL using the operational definitions shown in Box 1.

**Box 1. Operational definitions of Cutaneous Leishmaniasis (CL)**

•    CL is diagnosed in a person with skin and/or mucosal lesions with evidence of *Leishmania* infection in the affected tissues characterised by the presence of *Leishmania* amastigotes on smear microscopy or growth of *Leishmania* promastigotes in culture or the detection of *Leishmania* DNA by polymerase chain reaction.
•    Localized CL: A confirmed case of leishmaniasis, with no mucosal involvement, characterized by ten or fewer cutaneous papules and/or nodules and/or plaques with or without ulceration involving one body site
•    Multi-regional localized CL: A confirmed case of leishmaniasis, with no mucosal involvement, characterized by ten or fewer cutaneous papules and/or nodules and/or plaques with or without ulceration involving two or more body sites
•    Mucocutaneous leishmaniasis: A confirmed case of leishmaniasis characterized by ten or fewer papules and/or nodules and/or plaques with or without ulceration involving skin and an adjacent mucosal surface
•    Mucosal leishmaniasis: A confirmed case of leishmaniasis characterized by papules and/or nodules and/or plaques with or without ulceration involving exclusively a mucosal surface
•    Diffuse CL: A confirmed case of leishmaniasis characterized by eleven or more papules and/or nodules and/or plaques with or without mucosal involvement.

Body sites are classified as:

1. Head and neck

2. Torso - anterior (including genitalia)

3. Torso - posterior (including buttocks)

4. Right upper limb

5. Left upper limb

6. Right lower limb

7. Left lower limb

### Overview of data collection

Study visits for data collection are scheduled at day 1, 14, 28, 42, 90, 180 and 360 ( Table 1). Treatment will be decided by the treating clinicians, who may or may not be one of the study dermatologists. All study specific data collection will be undertaken by a member of the study team. At the baseline visit, we will collect socio-demographic data, perform clinical assessment, and collect data on PROMs and costs. Follow-up data will be collected for all patients at specified time points, as detailed further below. In a subset of patients, additional biological samples will be collected for immunological and genetic studies. A further subset of participants will be selected for in-depth interviews.

**Table 1.** Timetable of planned reviews and activities.. PSOAS-2: Patient and Observer Scar Assessment Scale version 2; DLQI: Dermatology Life Quality Index; CDLQI: Children’s Dermatology Life Quality Index; CLIQ: Cutaneous Leishmaniasis Impact Questionnaire; HLA: Human Leucocyte Antigen; PCR: polymerase chain reaction

	Day 1 (before 1 ^st^ treatment)	Day 14	Day 28 (end of 1 ^st^ cycle of systemic treatment)	Day 42	Day 90	Day 180	Day 360
**Participant and physician assessment of change**		X	X	X	X	X	
**Adverse reaction monitoring (if on treatment)**		X	X	X	X		
**Pain assessment**	X	X	X				
**Test of cure (if not healed)**					X		
**Scar assessment (PSOAS-2)**				X	X	X	X
**DLQI/CDLQI**	X	X	X	X	X	X	
**EQ-5D-5L**	X	X	X	X	X	X	X
**SF36**	X			X	X	X	X
**CLIQ**	X			X	X	X	
**Household costs**	X	X	X	X	X	X	X
**Immunological studies blood sample (subset)**	X		X		X		
**HLA typing blood sample**	X						
**Slit-skin smear for PCR**	X				X		
**Skin biopsy (subset of adults only)**	X				X		
**Digital image**	X	X	X	X	X	X	X

### Clinical data

At the baseline visit, we will record the clinical phenotype of CL including the number, size, morphology ^25^ and location of lesions. Lesion dimensions (defined as the largest diameter of the lesion) will be measured using a disposable tape measure. Individuals with more than one lesion will be asked to indicate the lesion causing greatest concern and this will be designated the “index” lesion. Individuals may indicate two or more lesions of equal concern. In those with mucosal involvement, mucosal signs and symptoms are recorded. Clinicians will then provide a subjective global assessment of CL severity using a 3-point Likert scale (mild, moderate, severe). Slit-skin smears will be collected at baseline for microscopy and species identification using polymerase chain reaction (PCR).

At each follow-up visit, we will assess lesions for the degree of re-epithelization or flattening. For those with DCL, the number of resolved lesions and percent of lesions flattened per region is recorded. Clinicians will provide a global assessment of the improvement or worsening of CL using a 5-point Likert scale (worse, no change, minor improvement, major improvement, cure). At each visit, we will record the treatment being administered and any changes since the last visit. The adverse reactions to CL treatments will be assessed including the nature and severity of the reaction, the action taken, the presumed cause and the outcome. The Common Terminology Criteria for Adverse Events (CTCAE) version 5.0 will be used for describing adverse reactions and grading their severity. Slit-skin smear samples will be collected at day 90 for test of cure if the skin lesion is not healed.

### PROMs

At baseline, Amharic or Oromiffa translations of several PROMs will be collected ( Table 1): two general measures of HRQoL, the EQ-5D-5L and the SF-36, and two skin-specific measures of HRQoL, the Dermatology Life Quality Index (DLQI) and the Children’s Dermatology Life Quality Index (CDLQI). Participants will complete the Cutaneous Leishmaniasis Impact Questionnaire (CLIQ), a CL-specific measure of HRQoL.

At the follow-up visits indicated in Table 1, we will collect PROMs again and ask participants for their global assessment of the improvement of their CL lesion(s) using a 5-point Likert scale (better, somewhat better, same, somewhat worse, worse). The Amharic or Oromiffa translations of the Patient and Observer Scar Assessment Scale (POSAS) v2 ^26^ will be completed by both the clinician and participant at the day 42 assessment and each subsequent visit.

### Costs

At baseline data will be collected on costs incurred by the individuals and their household that can be attributed to a member having CL and/or seeking treatment for CL including costs incurred since onset of CL symptoms. We will also collect data on household income, expenditure, and asset possession to establish a month of income and expenditure data.

At each follow up visit, cost related to CL since the last visit/admission will be collected. These will include costs of medicines, hospitalisation, consultation, transportation, and opportunity costs related to having and treating CL (i.e., missed work or school). We will also collect information on how households cope with the economic burden of CL.

Data from the provider perspective will be collected. This will include costs of training health workers, providing services, medicines donated, and other service-related costs. These costs will be collected from the project’s activity and financial records; the value of any donated items will be estimated based on market prices. Where necessary, government and donor sources will be consulted for relevant cost data.

### Lived experience

Participants will be recruited to participate in an in-depth interview to capture their lived experience as individuals with CL. Data will be collected from different age groups, gender, CL phenotype and places to ensure all variations to the level of saturation, where there is no new data with additional interviews. To reach saturation, an estimated 30 adults and 10 children interviews are planned. Interviews will be conducted by members of the research team using a topic-guide covering key themes that includes how it feels living with CL, impact of the illness on the person, the care-seeking pathways followed and their experience of traditional and modern health care service use. Participant observation will be used to enrich the data on patients' treatment experiences in the health facilities.

### Immunology samples

To interrogate the dynamics of immune responses in different clinical phenotypes of CL, blood and skin lesion samples will be collected at three time points. Whole blood and dried blood spot (DBS) samples will be collected by trained phlebotomists using the PAXgene® Blood DNA and RNA tubes and 3MM DBS filter papers. Physicians will take a skin biopsy samples from each CL cohort patient on the border site of the lesion using 3MM diameter disposable punches. HLA typing will be performed to determine if there is an association of HLA type with the clinical phenotypes of CL seen in our cohort. Participants will have a skin biopsy (stored in RNA *later*™) and blood samples taken to evaluate (i) the expression of innate and adaptive, and pro-and anti-inflammatory biomarkers using Luminex multiplex assay, (ii) changing mRNA expression level of Toll-like receptors (TLRs) and pro- and anti-inflammatory cytokines during infection, treatment and after completion of treatment using quantitative PCR (Real-time PCR).

### Data analysis

***Objective 1: To assess clinical outcomes in LCL, MCL and DCL in a prospective cohort.***

The primary outcome is the proportion of individuals with a healed index lesion by study day 90. In line with the consensus document on clinical trial outcomes cure will be defined as 100% re-epithelialization or flattening of the index lesion. Pre-specified additional outcomes are the proportion healed at day 42 and 180. Secondary outcomes include the frequency of adverse events and measures of HRQoL life.

There are no pre-existing high-quality data on cure rates for CL caused by *L. aethiopica*. We calculated the sample size required to measure a range of cure-rates at 90 days with varying degrees of precision ( Table 2). Based on this we aimed to recruit up to 250 participants with each CL phenotype (LCL, MCL, and DCL) across the two sites. We anticipate that mucosal leishmaniasis will be rare and will recruit as many individuals with this type of disease as possible.

**Table 2.** Sample sizes required to detect a range of cure rates with varying precision at day 90.

**Sample size**		**Percentage cured at 90 days**			
		50	60	70	80
	50	±13.1	±13.6	±12.7	±11.1
	100	±9.8	±9.6	±9.0	±7.8
	150	±8.0	±7.8	±7.3	±6.4
	200	±6.9	±6.8	±6.4	±5.5
	250	±6.2	±6.1	±5.7	±5.0

***Objective 2: To assess clinical parameters in LCL, MCL and DCL in a prospective cohort.***

We will use descriptive statistics (means, medians, and proportions) to describe demographics and baseline characteristics of the cohort. In exploratory analysis, we will use univariable logistic regression to examine clinical and demographic variables associated with resolution of CL at Day 42, 90, and 180. Whilst not formally powered for these analyses, the findings will be viewed as hypothesis-generating and potentially form the basis for future studies.

***Objective 3: To assess the change in HRQoL associated with CL before and after treatment.***

Changes in general, skin and CL-specific HRQoL will be assessed using the following PROMs: EQ-5D-5L, SF-36, DLQI/ CDLQI and CLIQ applied at the timepoints shown in Table 1, and compared with clinical outcomes to determine validity and responsiveness.

***Objective 4: To develop a severity scale for CL.***

A round table CL expert discussion forum, informed by preliminary data, will be held, and consensus reached about potential items and scoring system for a severity scale. The prototype will be tested in individuals with CL at diagnosis.

***Objective 5: To understand the lived experiences of individuals with CL.***

Recordings will be transcribed and translated verbatim, and interviewer’s notes included for each interview. A codebook will be developed, and thematic analysis will be used.

***Objective 6: To assess household costs of CL, and health care provider, and societal costs of treating CL.***

We will assess costs from a disaggregated societal perspective, examining costs to providers (MOH, donors), households, and society as a whole (i.e., costs to providers and household combined). Incremental costs to providers will be estimated relative to a hypothetical counterfactual in which no treatment for CL is provided. Incremental costs to households will be estimated relative to a hypothetical counterfactual in which the CL infection has not occurred. We will estimate total costs of diagnostic and treatment strategies and average costs, including cost per case diagnosed and cost per case resolved, disaggregated by site, type of CL, and patient characteristics (where possible), with appropriate statistical measures of variation. We will explore the degree of impoverishing and catastrophic expenditure for families of individuals with CL.

***Objective 7: To characterise how cell-mediated immune responses associate with treatment responses in CL due to L. aethiopica and (HLA) variation correlate with different clinical phenotypes.***

Biopsies of affected skin and blood samples will be analysed to (i) evaluate secretion and quantification of selected host cytokines via the LuminexⓇ platform, (ii) evaluate the expression of innate/adaptive, and pro-and anti-inflammatory biomarkers and by evaluating the change in mRNA expression level of TLRs and pro- and anti-inflammatory cytokines before treatment, during treatment, and after completion of treatment.

## Data management

Each participant will be assigned a unique study identification number and all data will be recorded and analysed with this unique identification number. Data will be electronically entered into a GCP-compliant, trial-specific study database [Research Electronic Data Capture (RedCap), Nashville, TN, USA]. Access to the database will be restricted to relevant researchers within the study team. Relevant documentation will be kept for a minimum of 10 years. Moreover, MAXQDA plus 2022 will be used to manage the in-depth interview data.

## Ethics

Ethical approval was obtained from AHRI/ALERT Ethics Review Committee (Ref: PO/23/21 dated 15.07.2021), the National Research Ethics Review Committee (Ref: 7/2-506/m259/35 dated 17.12.2021), and the London School of Hygiene and Tropical Medicine (Ref: 26421 dated 11.10.2021). Written individual informed consent will be obtained from all participants in the study (see *Extended data* ^27^). For study participants under 18 years of age, consent from a responsible adult (parent, relative, guardian, legal representative) will be obtained. In addition, assent will be obtained from study participants aged 12 to 17 years.

## Discussion

CL represents a major public health, psychosocial and economic problem both in Ethiopia and in the tropical – subtropical regions. A key target of the 2021-2030 WHO Neglected Tropical Diseases roadmap is a significant increase in the proportion of individuals with CL who are diagnosed and receive appropriate treatment. The 2021-25 Ethiopian strategic plan for Neglected Tropical Diseases, aims to meet the 2030 global target by increasing CL treatment centres from 14 to 30, diagnostic centres from 25 to 170 and to be able to detect 85% of cases and treat 95% of these. A better understanding of strategies to improve accessibility and treatment outcomes is a priority action to achieve this goal ^28^.

There are currently few prospective data of well-defined treatment outcomes for individuals with CL caused by *L. aethiopica*, and no data on PROMs or costs. A large cohort of participants with CL will generate robust data on how outcomes are associated with key clinical characteristics such as lesion size, site and number. This will allow us to gain insights into expected clinical outcomes up to one year following treatment and accurately quantify the burden of adverse reactions associated with current treatments. There are new agents and combination therapies proposed for the treatment of leishmaniasis and our data will be valuable in helping design future trials of these approaches in individuals with *L. aethiopica*.

This prospective cohort study will generate the most comprehensive and holistic dataset of CL, combining clinician determined and patient reported outcomes alongside health economic and laboratory data to better capture the impact of CL for affected individuals, their families, and communities. These data will inform the design of randomised trials to evaluate new treatment strategies, help improve evidence-based guidelines and support evidence-led policy decisions.

## Data Availability

No underlying data are associated with this article. LSHTM Data Compass: Prospective observational cohort study of cutaneous leishmaniasis in Ethiopia - Informed consent form. https://doi.org/10.17037/DATA.00003618 ^27^. Data are available under the terms of the Creative Commons Attribution 4.0 International license (CC-BY 4.0).

## References

[1] de Vries HJC Reedijk SH Schallig HDFH : Cutaneous Leishmaniasis: Recent Developments in Diagnosis and Management. *Am J Clin Dermatol.* 2015;16(2):99–109. 10.1007/s40257-015-0114-z 25687688 PMC4363483

[2] Yanik M Gurel MS Simsek Z : The psychological impact of cutaneous leishmaniasis. *Clin Exp Dermatol.* 2004;29(5):464–7. 10.1111/j.1365-2230.2004.01605.x 15347324

[3] Galvão EL Pedras MJ Cota GF : Development and initial validation of a cutaneous leishmaniasis impact questionnaire.Verdonck K, editor. *PLoS One.* 2018;13(8): e0203378. 10.1371/journal.pone.0203378 30161222 PMC6117079

[4] Salimi M Saghafipour A Parsa HH : Economic Burden Evaluation of Cutaneous Leishmaniasis in Iran. *Shiraz E-Medical J.* 2019;20(6): e82810. 10.5812/semj.82810

[5] Wijerathna T Gunathilaka N Gunawardena K : The Economic Impact of Cutaneous Leishmaniasis in Sri Lanka. *Biomed Res Int.* 2018;2018: 3025185. 10.1155/2018/3025185 30406132 PMC6201334

[6] Walayat Hussain TS Motley RJ Wang TS : Cryosurgery. In: Griffiths CEM, Barker J, Bleiker TO, Chalmers R, Creamer D, editors. *Rook’s Textbook of Dermatology*. John Wiley & Sons, 2016;2016.

[7] Aronson N Herwaldt BL Libman M : Diagnosis and treatment of leishmaniasis: Clinical practice guidelines by the infectious diseases society of America (IDSA) and the American Society of tropical medicine and hygiene (ASTMH). * Am J Trop Med Hyg.* 2017;96(1):24–45. 10.4269/ajtmh.16-84256 27927991 PMC5239701

[8] De Vries HJC Schallig HD : Cutaneous Leishmaniasis: A 2022 Updated Narrative Review into Diagnosis and Management Developments. *Am J Clin Dermatology.* 2022;23(6):823–840. 10.1007/s40257-022-00726-8 36103050 PMC9472198

[9] van Henten S Adriaensen W Fikre H : Cutaneous Leishmaniasis Due to *Leishmania aethiopica*. *EClinicalMedicine.* 2018;6:69–81. 10.1016/j.eclinm.2018.12.009 31193672 PMC6537575

[10] Fikre H Mohammed R Atinafu S : Clinical features and treatment response of cutaneous leishmaniasis in North-West Ethiopia. *Trop Med Int Heal.* 2017;22(10):1293–1301. 10.1111/tmi.12928 28712122

[11] van Griensven J Gadisa E Aseffa A : Treatment of Cutaneous Leishmaniasis Caused by *Leishmania aethiopica*: A Systematic Review. *PLoS Negl Trop Dis.* 2016;10(3): e0004495. 10.1371/journal.pntd.0004495 26938448 PMC4777553

[12] Akuffo H Dietz M Teldemariam S : The use of itraconazole in the treatment of leishmaniasis caused by *Leishmania aethiopica*. *Trans R Soc Trop Med Hyg.* 1990;84(4):532–4. 10.1016/0035-9203(90)90028-d 1965355

[13] Van Der Meulen J Mock B Fekete E : Limited therapeutic action of rifampicin/isoniazid against Leishmania aethiopica.Ltd Ther action rifampicin/isoniazid against Leishmania aethiopica.1981;318(8239):197–8. 10.1016/S0140-6736(81)90374-3 6114258

[14] Negera E Gadisa E Hussein J : Treatment response of cutaneous leishmaniasis due to *Leishmania aethiopica* to cryotherapy and generic sodium stibogluconate from patients in Silti, Ethiopia. *Trans R Soc Trop Med Hyg.* 2012;106(8):496–503. 10.1016/j.trstmh.2012.02.006 22503475

[15] Padovese V Terranova M Toma L : Cutaneous and mucocutaneous leishmaniasis in Tigray, northern Ethiopia: clinical aspects and therapeutic concerns. *Trans R Soc Trop Med Hyg.* 2009;103(7):707–11. 10.1016/j.trstmh.2009.02.023 19356780

[16] Heras-Mosteiro J Monge-Maillo B Pinart M : Interventions for Old World cutaneous leishmaniasis. * Cochrane Database Syst Rev.* John Wiley and Sons Ltd;2017;2017(11): CD005067. 10.1002/14651858.CD005067.pub4 29149474 PMC6486265

[17] Olliaro P Grogl M Boni M : Harmonized clinical trial methodologies for localized cutaneous leishmaniasis and potential for extensive network with capacities for clinical evaluation. Satoskar AR, editor. *PLoS Negl Trop Dis.* 2018;12(1): e0006141. 10.1371/journal.pntd.0006141 29329311 PMC5785032

[18] Zewdu FT Tessema AM Zerga AA : Effectiveness of intralesional sodium stibogluconate for the treatment of localized cutaneous leishmaniasis at Boru Meda general hospital, Amhara, Ethiopia: Pragmatic trial. *PLoS Negl Trop Dis.* 2022;16(9): e0010578. 10.1371/journal.pntd.0010578 36084153 PMC9491591

[19] van Henten S Tesfaye AB Abdela SG : Miltefosine for the treatment of cutaneous leishmaniasis— A pilot study from Ethiopia. *PLoS Negl Trop Dis.* 2021;15(5): e0009460. 10.1371/journal.pntd.0009460 34048461 PMC8191986

[20] Reithinger R Coleman PG : Treating cutaneous leishmaniasis patients in Kabul, Afghanistan: cost-effectiveness of an operational program in a complex emergency setting. *BMC Infect Dis.* 2007;7: 3. 10.1186/1471-2334-7-3 17263879 PMC1790896

[21] Stahl HC Ahmadi F Nahzat SM : Health economic evaluation of moist wound care in chronic cutaneous leishmaniasis ulcers in Afghanistan. *Infect Dis Poverty.* 2018;7(1): 12. 10.1186/s40249-018-0389-4 29444705 PMC5812215

[22] Refai WF Madarasingha NP Sumanasena B : Efficacy, Safety and Cost-Effectiveness of Thermotherapy in the Treatment of *Leishmania donovani* - Induced Cutaneous Leishmaniasis: A Randomized Controlled Clinical Trial. *Am J Trop Med Hyg.* 2017;97(4):1120–1126. 10.4269/ajtmh.16-0879 28820681 PMC5637590

[23] Malik F Hanif MM Mustafa G : Comparing the efficacy of oral chloroquine *versus* oral tetracycline in the treatment of cutaneous leishmaniasis. * J Coll Physicians Surg Pak.* 2019;29(5):403–405. 10.29271/jcpsp.2019.05.403 31036105

[24] Federal Ministry of Health- Ethiopia: Guideline for the diagnosis, treatment and prevention of leishmaniasis in Ethiopia. Addis Abeba;2013. Reference Source

[25] Nast A Griffiths CEM Hay R : The 2016 International League of Dermatological Societies’ revised glossary for the description of cutaneous lesions. *Br J Dermatol.* 2016;174(6):1351–8. 10.1111/bjd.14419 26801523

[26] POSAS - The Patient and Observer Scar Assessment Scale. Reference Source

[27] Lambert S : Prospective observational cohort study of cutaneous leishmaniasis in Ethiopia - Informed consent form.[Data Collection]. London School of Hygiene & Tropical Medicine, London, United Kingdom, 2023. 10.17037/DATA.00003618

[28] Ending the neglect to attain the Sustainable Development Goals: A road map for neglected tropical diseases 2021–2030.[cited 2023 Mar 12]. Reference Source

