# Supplementary material for: Protocol for a prospective observational cohort study of cutaneous leishmaniasis in Ethiopia
Source: NIHR Open Res. 2023 Oct 5;3:49. [Version 1] doi: 10.3310/nihropenres.13432.1 (PMC11748424; doi:10.3310/nihropenres.13432.1)
Supplement: Peer review history

# Reviewer report: Reviewer response for version 1

**DOI:** 10.3310/nihropenres.14569.r30757

**Published:** 2023-11-23

**Authors:** Talal Alharazi

**Affiliations:** 1 Taiz University, Taiz, Yemen

**Introduction**

- Shorten the background/introduction to be more concise for a quantitative study

**Methods**

- Expand on the inclusion/exclusion criteria, especially related to prior treatments
- Specify which cytokines/biomarkers will be analyzed in the immunology studies
- Elaborate on the sample size calculations and precision expected
- State which statistical tests and analysis software will be used

**Analysis Plan**

- Specify proposed statistical tests and significance levels for objectives

**Discussion **

- Reduce repetition of content covered elsewhere

**References**

- Update older references, over 5 years old unless seminal works

Overall, add more methodological specifics in line with quantitative reporting guidelines to facilitate reproducibility and interpretation.

Is the study design appropriate for the research question?

Partly

Is the rationale for, and objectives of, the study clearly described?

Yes

Are sufficient details of the methods provided to allow replication by others?

Partly

Are the datasets clearly presented in a useable and accessible format?

Not applicable

Reviewer Expertise:

My work primarily focuses on the epidemiology and control of malaria and neglected tropical diseases, including schistosomiasis, leishmaniasis, and other protozoan and helminth parasites .Also conducts research on the epidemiology of microorganisms and multidrug-resistant bacteria.

I confirm that I have read this submission and believe that I have an appropriate level of expertise to confirm that it is of an acceptable scientific standard, however I have significant reservations, as outlined above.

# Response

**Published:** 2024-12-01

**Authors:** Saba Lambert

**Affiliations:** Clinical Research, London School Hygiene and Tropical Medicine, LOndon, UK

Dear Dr Alharazi,

 We are grateful for your careful review of our protocol paper and your comments are important in helping us to improve it. We are addressing these one by one here.

**Introduction**

- Shorten the background/introduction to be more concise for a quantitative study

**It is mixed method research.**

**This introduction reflects what is known about CL in Ethiopia and what data is available so far, bringing to the fore questions that need to be addressed. In view of the complex multi-disciplinary issues being addressed, and to address CL In Ethiopia holistically, we believe the introduction is as concise and inclusive as possible.**

**Methods**

- Expand on the inclusion/exclusion criteria, especially related to prior treatments

**Thank you, we have elaborated these criteria as follows:**

**“All adults and children diagnosed with CL and initiated on CL treatment at both study centers are eligible to be recruited in this study. Each participant will be followed for 360 days.**

**Inclusion criteria: **

1. **Individuals with parasitologically confirmed CL using microscopy or culture **
2. **Individuals receiving treatment at the study centers**

**Exclusion criteria: **

1. **Participants who have previously been treated with systemic anti-leishmania therapy**
2. **Participants who do not or are unable to give informed consent** **to participate in the study”**

 

- Specify which cytokines/biomarkers will be analyzed in the immunology studies

**We have amended the text to:**

**“To interrogate the dynamics of immune responses in different clinical phenotypes of CL, blood and skin lesion samples will be collected at three time points. Participants will have blood samples and a skin biopsy (stored in RNAlater™) taken to evaluate subsets of the innate and adaptive immunity; pro-inflammatory (IFN-γ and TNF-α) and anti-inflammatory (IL-6, IL-27, IL-10, IL-4, IL-13, TGF-β, and hsp18) cytokines and interleukins (IL)-12, IL-2, IL-1, IL-18, IL-15, IL-8, IL-17A, and IL-22) using Luminex multiplex assay. (ii) The change in mRNA (Ribonucleic acid) expression level of Toll-like receptors (TLRs) and pro-inflammatory (IFN-γ, IL-12, IL-1β, IL-17A, and IL-12p70) and anti-inflammatory (IL-5, IL-6, IL-10, IL-4, TGF-β, and hsp18) cytokines will also be assessed before treatment (at day 0), during treatment (day 28) and after completion of treatment (day 90) using quantitative PCR (Real-time Polymerase Chain Reaction). In addition, the levels of chemokines (such as CXCL8, CXCL10, CCL5, CCL4, CCL3, and CCL2) will be determined in supernatants from the whole blood stimulation assays for the Luminex multiplex assay. HLA typing will be performed to assess if there is an association between HLA types (targeting probes for HLA-A, B, C, DR, DQ, and DP loci) and the clinical phenotypes of CL seen in our cohort.”**

  

- Elaborate on the sample size calculations and precision expected
** Table 2 details the sample sizes required for different cure rates.**

 

- State which statistical tests and analysis software will be used

**This has now been under in each section of the Data Analysis paragraph**

**Analysis Plan**

- Specify proposed statistical tests and significance levels for objectives

**This has now been under in each section of the Data Analysis paragraph**

**Discussion**

- Reduce repetition of content covered elsewhere

**We feel the brief discussion summarizes the important points presented previously.**

**References**

- Update older references, over 5 years old unless seminal works

**CL is a NTD and research in Ethiopia is limited. We believe we have provided a thorough literature review. There are a few more recent articles, but these were not available at the date of our protocol development. These will be reviewed when results are presented.**

  

- Overall, add more methodological specifics in line with quantitative reporting guidelines to facilitate reproducibility and interpretation.

**We believe our paper contains detailed methodological description**

 Thank you for helping us improve our paper

# Reviewer report: Reviewer response for version 1

**DOI:** 10.3310/nihropenres.14569.r30538

**Published:** 2023-11-01

**Authors:** Najia Ahmed

**Affiliations:** 1 Dermatology, Pakistan Naval Station Shifa Hospital, Karachi, Sindh, Pakistan; 2 Bahria University of Health Sciences, Karachi, Pakistan

- The study design has been stated as prospective observational cohort but it appears to be a mixed method study as it also involves patient interviews and their experiences.
- Introduction is too long if it is supposed to be quantitative research.
- Inclusion and exclusion criteria need to be elaborated more.
- In immunology samples, expressions of innate and adaptive immunity and pro and anti inflammatory cytokines to be measured, have not been specified.
- Explain in detail how sample size was calculated.
- Give details of software to be used for data analysis, statistical tests and significant values that will be used.
- Some of the abbreviations have also not been explained.
- Repetition of content in discussion.
- 19 of the 28 ref used are more than 5 years old.
- Ref 26, not in Vancouver style. 
- No PMID or DOI for ref provided.

Is the study design appropriate for the research question?

Partly

Is the rationale for, and objectives of, the study clearly described?

Yes

Are sufficient details of the methods provided to allow replication by others?

Partly

Are the datasets clearly presented in a useable and accessible format?

Not applicable

Reviewer Expertise:

Section of data analysis, specially the tests of significance to be used, should also be assessed by a statistician.

I confirm that I have read this submission and believe that I have an appropriate level of expertise to confirm that it is of an acceptable scientific standard, however I have significant reservations, as outlined above.

# Response

**Published:** 2024-12-01

**Authors:** Saba Lambert

**Affiliations:** Clinical Research, London School Hygiene and Tropical Medicine, LOndon, UK

RESPONSE TO COMMENTS:

 Dear Dr Ahmed

 We are grateful for your careful review of our protocol paper and your comments are important in helping us to improve it. We are addressing these one by one here.

  

- The study design has been stated as prospective observational cohort but it appears to be a mixed method study as it also involves patient interviews and their experiences.

**Indeed, this has been designed as a prospective observational cohort into which are integrated different methodologies appropriate to the different “areas” under investigation, using qualitative and quantitative tools:**

  

- **Clinical: Observation of clinical features, treatments given, clinical outcomes as well as patient reported outcome measure (quantitative)**
- **Psychosocial: patient’s treatment experience using ethnographic methods; qualitative (IDI)**
- **Immunological investigations on skin and blood samples – quantitative laboratory research**
- **Economic burden of CL: Serial economic evaluations**

** We have added a brief clarification on page 6 under “Overview of Data Collection”**

  

- Introduction is too long if it is supposed to be quantitative research.

**We employ a variety of methods, and the length of the introduction reflects the broad   scope of this cohort study.**

**This introduction reflects what is known about CL in Ethiopia and what data is available so far, bringing to the fore questions that need to be addressed. In view of the complex multi-disciplinary issues being addressed, and to address CL In Ethiopia holistically, we believe the introduction is as concise and inclusive as possible.**

- Inclusion and exclusion criteria need to be elaborated more.

**Thank you, we have elaborated these criteria as follows:**

**“All adults and children diagnosed with CL and initiated on CL treatment at both study centers are eligible to be recruited in this study. Each participant will be followed for 360 days.**

**Inclusion criteria: **

1. **Individuals with parasitologically confirmed CL using microscopy or culture **
2. **Individuals receiving treatment at the study centers**

**Exclusion criteria: **

1. **Participants who have previously been treated with systemic anti-leishmania therapy**
2. **Participants who do not or are unable to give informed consent** **to participate in the study**

- In immunology samples, expressions of innate and adaptive immunity and pro and anti inflammatory cytokines to be measured, have not been specified.

**We have amended the text to:**

**“To interrogate the dynamics of immune responses in different clinical phenotypes of CL, blood and skin lesion samples will be collected at three time points. Participants will have blood samples and a skin biopsy (stored in RNAlater™) taken to evaluate subsets of the innate and adaptive immunity; pro-inflammatory (IFN-γ and TNF-α) and anti-inflammatory (IL-6, IL-27, IL-10, IL-4, IL-13, TGF-β, and hsp18) cytokines and interleukins (IL)-12, IL-2, IL-1, IL-18, IL-15, IL-8, IL-17A, and IL-22) using Luminex multiplex assay. (ii) The change in mRNA (Ribonucleic acid) expression level of Toll-like receptors (TLRs) and pro-inflammatory (IFN-γ, IL-12, IL-1β, IL-17A, and IL-12p70) and anti-inflammatory (IL-5, IL-6, IL-10, IL-4, TGF-β, and hsp18) cytokines will also be assessed before treatment (at day 0), during treatment (day 28) and after completion of treatment (day 90) using quantitative PCR (Real-time Polymerase Chain Reaction). In addition, the levels of chemokines (such as CXCL8, CXCL10, CCL5, CCL4, CCL3, and CCL2) will be determined in supernatants from the whole blood stimulation assays for the Luminex multiplex assay. HLA typing will be performed to assess if there is an association between HLA types (targeting probes for HLA-A, B, C, DR, DQ, and DP loci) and the clinical phenotypes of CL seen in our cohort.”**

  

- Explain in detail how sample size was calculated.

**Table 2 details the sample sizes required for different cure rates.**


**  **

- Give details of software to be used for data analysis, statistical tests and significant values that will be used.

**The following have been expanded in each section**

**Clinical: Data will be analysed in STATA. Descriptive statistics will be used to describe the cohort. Odds ratio and logistic regression models will be used to identify clinical and demographic characteristics associated with healing.**

**PROMS: Validity and responsiveness of each PROM will be analysed using SPSS version 23.0 (IBM Corporation, Armonk, NY, USA). Descriptive statistics will be presented using frequencies, percentages, tables, and graphs.**

**Mean rank sum scores of each of sub-domains will be recorded using Kruskal Wallis rank sum test among as well as the different clinical (LCL, MCL and DCL) types of lesions to check known-group construct validity. Wilcoxon-signed rank test will be used to compare mean difference of scores before initiation of treatment (Day 0) and at Day 90 (date of initial cure assessment).**

**Laboratory: Statistical analyses of laboratory data will be performed using R (version 4.4.1, the R Foundation for Statistical Computing). Nonparametric Mann-Whitney statistics will be used to compare different groups. Differences in cytokine and chemokine concentrations and mRNA expressions among CL types, treatment outcomes, time points, and other variables will be compared by the Kruskal-Wallis and t-tests. Fisher's exact test will be employed to determine the association between HLA types and CL types.**

**Economics: Descriptive statistics:  frequencies, percentages, means and medians will be used as appropriate to estimate costs. Further analysis will be conducted to investigate catastrophic out-of-pocket health expenditure and poverty impact using a linear probability model. STATA version 17 will be used for data analysis**

**In-depth interviews: Recordings will be transcribed and translated verbatim, and interviewer’s notes included for each interview. A codebook will be developed, and thematic analysis will be used. MAXQDA plus 2022 will be used to manage the in-depth interview data.**

  

- Some of the abbreviations have also not been explained.

**Thank you, this has been amended.**


**  **

- Repetition of content in discussion.

**We feel the brief discussion summarizes the important points presented previously.**


**  **

- 19 of the 28 ref used are more than 5 years old.

**CL is a NTD and research in Ethiopia is limited. We believe we have provided a thorough literature review. There are a few more recent articles, but these were not available at the date of our protocol development. These will be reviewed when results are presented.**


**  **

- Ref 26, not in Vancouver style. 
   
**We have checked referencing style. Thank you.**
- No PMID or DOI for ref provided.

**Apologies, this was provided but does not seem to have uploaded correctly. Will amend with editorial team**
